# Negative HBcAg in immunohistochemistry assay of liver biopsy is a predictive factor for the treatment of patients with nucleos(t)ide analogue therapy

**DOI:** 10.1111/jcmm.13444

**Published:** 2017-11-29

**Authors:** Mingxing Huang, Jian Liu, Monica Chow, Xuan Zhou, Zongping Han, Zhenjian He, Jinfang Xue, Zhe Zhu, Xinhua Li, Jinyu Xia

**Affiliations:** ^1^ Department of Infectious Diseases The Fifth Affiliated Hospital of Sun Yat‐Sen University (SYSU) Zhuhai Guangdong China; ^2^ PGY IV Department of General Surgery Rutgers Robert Wood Johnson Medical School Piscataway NJ USA; ^3^ School of Public Health Sun Yat‐sen University Guangzhou China; ^4^ Department of Medicine Division of Regenerative Medicine University of California San Diego School of Medicine La Jolla CA USA; ^5^ Department of Stem Cell Biology and Regenerative Medicine Cleveland Clinic Lerner Research Institute Cleveland OH USA; ^6^ Department of Infectious Diseases The Third Affiliated Hospital Sun Yat‐Sen University Guangzhou Guangdong China

**Keywords:** hepatitis B core antigen, hepatitis B e antigen, immunohistochemistry, chronic hepatitis B, nucleos(t)ide analogue

## Abstract

The hepatitis B core antigen (HBcAg) is an important target for antiviral response in chronic hepatitis B (CHB) patients. However, the correlation between HBcAg in the hepatocyte nucleus and nucleos(t)ide analogue (NA) therapeutic response is unclear. We sought to evaluate the role of HBcAg by analysing liver biopsies for viral response in NA‐naïve hepatitis B e antigen (HBeAg) positive (+) CHB patients *via* immunohistochemistry (IHC). A total of 48 HBcAg‐negative (−) patients and 48 HBcAg (+) patients with matching baseline characteristics were retrospectively analysed for up to 288 weeks. Virological response (VR) rates of patients in the HBcAg (−) group were significantly higher at week 48 and 96 than the HBcAg (+) group (77.1% *versus* 45.8% at week 48, respectively, *P *=* *0.002 and 95.3% *versus* 83.3% at week 96, respectively, *P *=* *0.045). The serological negative conversion rate of HBeAg was significantly higher in the HBcAg (−) than in the HBcAg (+) group from week 96 to 288 (35.4 % *versus* 14.6% at week 96, respectively, *P *=* *0.018; 60.4% *versus* 14.6%, respectively, *P *<* *0.001 at week 144; 72.9% *versus* 35.4%, respectively, *P *<* *0.001 at week 288). The cumulative frequencies of VR and lack of HBeAg were higher in the HBcAg (−) group (both *P* < 0.05). Binary logistic regression analysis showed that HBcAg (−) was the predictor for the lack of HBeAg (OR 4.482, 95% CI: 1.58–12.68). In summary, the absence of HBcAg in the hepatocyte nucleus could be an independent predictor for HBeAg seroconversion rates during NA‐naïve treatment in HBeAg (+) CHB patients.

## Introduction

Hepatitis B virus (HBV) is still a major global health problem and the leading cause of liver diseases, cirrhosis and hepatocellular carcinoma 1, with an estimate of 240 million patients worldwide 2. As a result, more than 686,000 people die every year due to complications caused by hepatitis B 1, 2, 3, 4.

Active replication of HBV is indicated by the expression of hepatitis B e antigen (HBeAg) 5, which is closely associated with an increased risk of hepatocellular carcinoma 6. Loss of HBeAg expression and/or neutralization of HBeAg by antibodies has been shown to be an end‐point for treatment related to chronic hepatitis B (CHB) 7, 8. However, in nucleos(t)ide analogue (NA)‐naïve CHB patients, the frequency of elimination of HBeAg expression is very low. It was reported that only 10% of NA‐naïve CHB patients on entecavir (ETV) at week 48, 21% at week 96 and 34% at week 144 9 had positive‐to‐negative seroconversion of HBeAg. For NA‐naïve CHB patients on tenofovir disoproxil fumarate (TDF), 23.6% and 40% had positive‐to‐negative seroconversion of HBeAg 72 weeks and 5 years, respectively, after treatment 10. CHB patients on a combined TDF and ETV treatment only had an 18% (125/138) and 21.7% (30/138) seroconversion rate at weeks 48 and 96, respectively 11.

Hepatitis B core antigen (HBcAg) is also known to be an important marker for the proliferation of HBV 12. HBcAg is coded by gene C of HBV, which is essential to viral replication 13. From immunohistochemical (IHC) staining patterns, HBcAg can be classified as having cytoplasmic expression (cHBcAg), nuclear expression (nHBcAg), cytoplasmic and nuclear‐mixed expression (mHBcAg) or negative expression 14. The different distributions of HBcAg expression in the hepatocyte nucleus and cytoplasm of CHB indicate the level of viral replication and histological activity 12, 13, 14. HBcAg is localized mainly in the nucleus in the viral replicative or tolerance stage and nucleus and/or cytoplasm of hepatocytes during the viral clearance phase 15, 16. Serum HBc antigen concentration is positively correlated with intrahepatic covalently closed circular DNA (cccDNA) levels 17. Therefore, HBcAg in the hepatocyte nucleus also may positively correlate with intrahepatic cccDNA levels, which could serve as another important predictor for virological response during antiviral therapy for CHB patients 18.

There have been limited reports detailing the role of hepatocyte nuclear HBcAg status in antiviral therapy. The aim of this study was to investigate the role of hepatocyte nuclear HBcAg in HBeAg‐positive (+) NA‐naïve CHB patients during NA therapy. We examined the viral response rates and the serum HBeAg‐negative (−) rates of HBcAg (−) and HBcAg (+) patients using IHC on liver biopsies.

## Materials and methods

### Patient selection

All the selected patients in our study group were from the 5th Affiliated Hospital, Sun Yat‐sen University (SYSU). The study was conducted in accordance with the guidelines of the Declaration of Helsinki and was approved by the 5th Affiliated Hospital Ethical Committee at SYSU. The study design and manuscript preparation fully followed the guidelines from the STROBE statement 19. Written informed consent was obtained from all patients.

From 1 January 2010 to 31 July 2016, 96 NA‐naïve CHB patients with serum positive for HBeAg were enrolled. For patients with HBV infection, parameters including age, sex, serum alanine aminotransferase (ALT), HBV DNA levels at baseline and throughout treatment, quantities of hepatitis B surface antigen (HBsAg), HBeAg and anti‐HBe status/levels, time to ALT normalization, time to undetectable HBV DNA levels and time to HBeAg (+) to (−) seroconversion during follow‐up were recorded for each patient prior to treatment.

Patients were excluded from this study if they (1) were serum HBeAg negative, (2) were coinfected with other hepatitis viruses or had other comorbidities, (3) displayed alcoholic, drug‐induced or autoimmune liver diseases, (4) were pregnant or lactating and (5) had been treated with NA previously.

**Figure 1:**
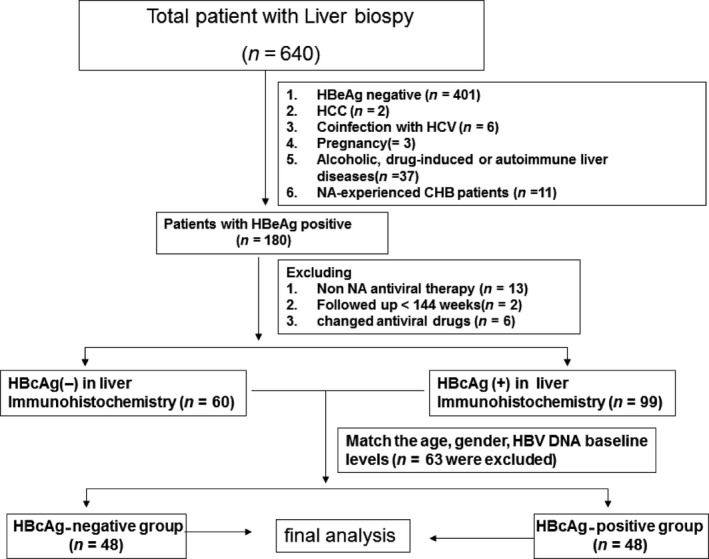
Flow chart of the patients selected for the respective research.

Patients were divided into two groups, HBcAg (−) and HBcAg (+), based on the lack of or presence of HBcAg in hepatocyte nucleus, respectively. A total of 48 HBcAg (−) patients and 48 HBcAg (+) patients matched in sex, age and baseline HBV DNA levels were included in this study (Table 1; Fig. 1). Histological inflammatory activity (determined by simple grading and staging systems for chronic viral or autoimmune hepatitis) 20 and IHC of liver biopsies were measured before starting NA therapy. Patients followed up every 3 to 6 months for blood collection and liver ultrasound examination for a total of 288 weeks. All the patients were treated according to the guidelines of prevention and treatment for CHB (2010 edition) 21.

**Table 1:** The baseline demography of the patients in HBcAg (−) and HBcAg (+) groups

Items	HBcAg (−) (*n* = 48)	HBcAg (+) (*n* = 48)	*P*
Age, year	37 (22–59)	35 (23–59)	0.712
Gender, M%	85.4 (41/48)	79.2 (38/48)	0.423
ALT, U/l	115.6 ± 157.6	170.2 ± 320.8	0.286
HBV DNA, log_10_ IU/ml	6.02 ± 1.27	6.28 ± 1.18	0.311
HBsAg, Log_10_ IU/ml	3.14 ± 0.61	3.09 ± 0.65	0.660
HBeAg, Log_10_ S/CO	1.82 ± 0.52	1.90 ± 0.75	0.476
HBcAb,‐Log10 S/CO	1.23 ± 0.14	1.18 ± 0.18	0.179
Knodell necroinflammatory score^a^			
Grade 1	4 (8.3%)	8 (16.7%)	
Grade 2	29 (60.4%)	31 (64.6%)	
Grade 3	15 (31.3%)	9 (18.7%)	0.280
Fibrosis^a^			
Stage 0	8 (16.7%)	10 (20.8%)	
Stage 1	8 (16.7%)	7 (14.6%)	
Stage 2	8 (16.7%)	9 (18.7%)	
Stage 3	7 (14.5%)	9 (18.7%)	
Stage 4	17 (35.4%)	13 (27.1%)	0.900

HBcAb reference: 1.0000–3.000 negative and 0–1.000 positive.

HBeAg reference: <1 negative and >1 positive.

HBeAb reference: <1 positive and >1 negative.

ALT: Alanine transaminase.

a Simple grading and staging systems for chronic viral hepatitis 24.

### NA therapy and detection methods

All the patients received daily NA therapy (including lamivudine (LAM), adefovir dipivoxil (ADV), telbivudine (LDT), entecavir (ETV) or ADV combined with either LAM or ETV). A liver biopsy was taken from each patient prior to treatment initiation. HBsAg and HBcAg in the hepatocyte nucleus were measured using IHC staining with mouse anti‐human HBsAg monoclonal antibody and rabbit anti‐human HBcAg polyclonal antibody, respectively. Staining was performed according to the manufacturer's protocols (Fuzhou Maixin Biotech. Co., Ltd. Fuzhou, China). Diagnosis was based on positive staining by the HBc antibody. The reports of liver histology were generated by a pathologist. Liver function (showed in the serum ALT, AST TBIL) and kidney function (showed in serum creatinine, urea nitrogen) were both tested by the machine of Hitachi 7180 (Hitachi, Ltd., Tokyo, Japan). Normal range of ALT value is 5–35 U/l. HBV DNA levels were measured by real‐time fluorescence quantitative polymerase chain reaction assays (Applied Biosystems, ABI 7500, the lowest limit of detection was 100 IU/ml). HBsAg, HBeAg, anti‐HBe and anti‐HBc were detected using Architect i2000SR (Abbott Laboratories). Hepatic inflammation was assessed using simple grading (score 1–4) and staging (score 0–4) systems for chronic viral hepatitis 20. In brief, the different stages of chronic liver diseases relate to the degree of scarring, with the end stage being cirrhosis. The grade relates to the severity of the underlying disease process. The histology activity index (HAI) is appropriate for evaluation of a large numbers of patients.

#### Definitions

**Figure 2:**
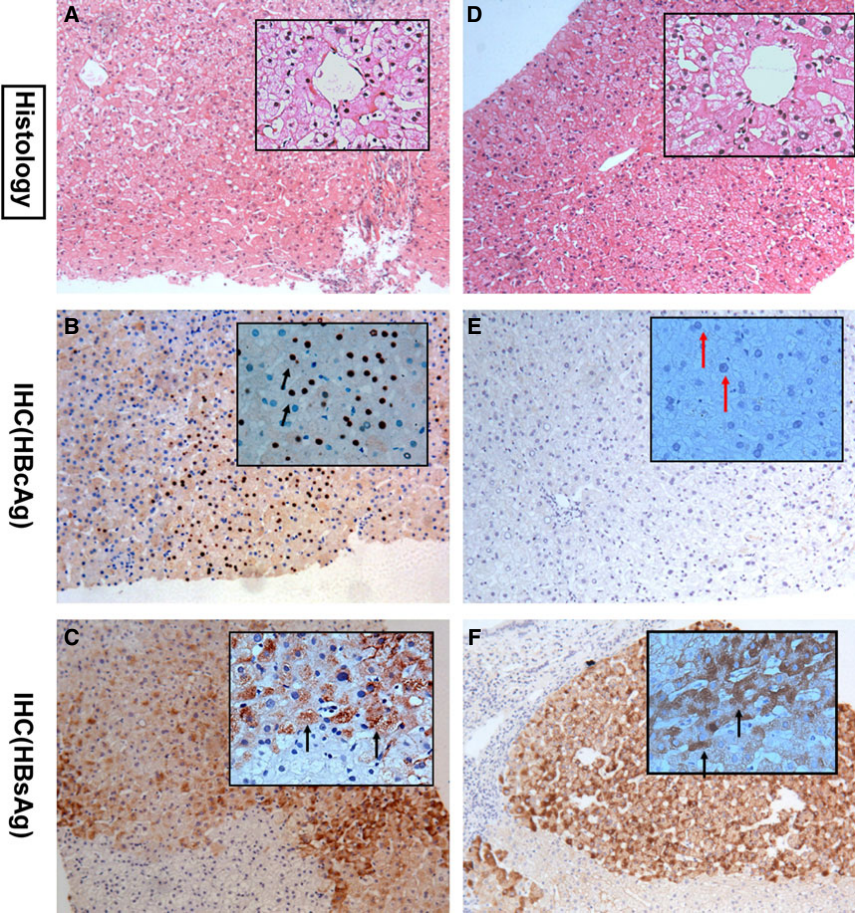
HBcAg in the hepatocytes of patients in HBcAg‐negative and HBcAg‐positive groups. Both case #1 (HBcAg‐negative group) and case #2 (HBcAg‐positive group) were diagnosed G2S1. H.E. staining in the case #1 (**A**) and case #2 (**D**); IHC with HBcAg were positive with black arrows (**B**) or negative with the red arrows (**E**) in the hepatocyte nucleus. HBsAg was positive in black arrows (**C**,** F**) in the hepatocytes.

HBcAg (−) or (+) was defined as the lack of or presence of HBcAg, respectively, in the hepatocyte nucleus on IHC staining of liver biopsies (Fig. 2). Virological response was defined as undetectable serum HBV DNA (<2.0 log_10_ IU/ml) or undetectable with qPCR assay. HBeAg (−) was defined as <1 s/co. HBeAg seroconversion was defined as HBeAb >1 and HBeAg <1 s/co. The value of 1.00 to 3.00 in HBcAb was defined as negative, while 0 to 1.00 was defined as positive.

### Statistical analyses

Appropriate statistical analysis was performed using Prism version 5 (GraphPad Software). Categorical variables were defined as proportion (%) and compared by chi‐square or Fisher's exact test. Continuous variables are depicted as mean ± standard deviation (SD) and were assessed by Student's *t*‐test or Mann–Whitney *U* test, as appropriate. Binary logistic regression analysis was performed in search of variables determining the HBeAg‐negative status. Cumulative rates of complete viral suppression and ALT normalization were analysed by the Kaplan–Meier method. *P *<* *0.05 was considered statistically significant.

## Results

### Patient characteristics

A total of 96 serum HBeAg (+) CHB patients were included in this study with 48 patients in the HBcAg (−) group and 48 patients in the HBcAg (+) group. They were all matched in age, sex, HBV DNA baseline, HBeAg levels, HBsAg levels, HBcAb level and ALT levels (Table 1). The liver biopsy IHC stain for HBcAg (−) and HBcAg (+) is shown in Fig. 2A–E. There were no significant differences either in Knodell necroinflammatory grade or in cirrhosis stage (*P *=* *0.280, 0.900, respectively.)

### Virological response

**Figure 3:**
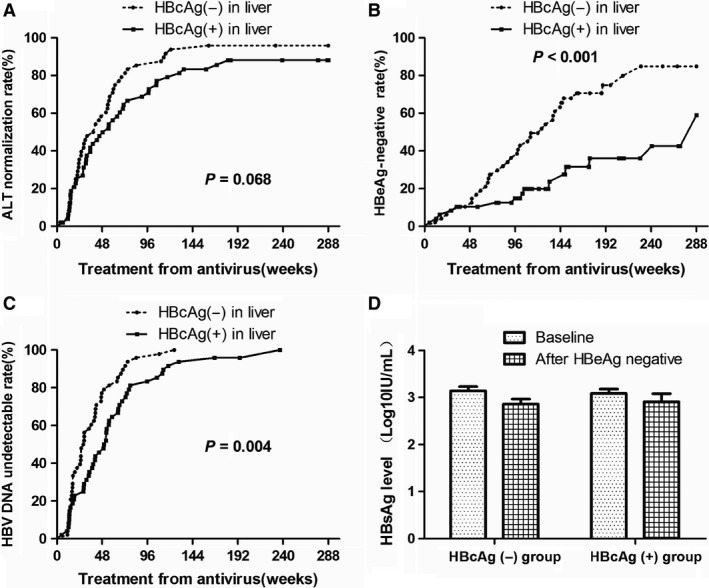
The Kaplan–Meier survival analysis of HBV DNA cumulative rates, ALT normalization cumulative rates (**A**) and HBeAg cumulative negative rates (**B**) and they were all significant difference in HBcAg (−) group (**C**) and HBcAg (+) group (**D**).

Compared with patients in the HBcAg (+) group, the frequency of patients with undetectable HBV DNA in the HBcAg (−) group was significantly higher at week 48 (77.1% *versus* 45.8%, *P* = 0.002) after starting of NA treatment and 96 (95.3% *versus* 83.3%, *P *=* *0.045; Table 2). In addition, Kaplan–Meier survival analysis revealed that significant differences were found in HBV DNA levels between the two groups (*P *=* *0.004) by 288 weeks. The median survival time of HBV DNA was at 28.4 and 51.0 weeks (Fig. 3A).

**Table 2:** All correlated factors of HBeAg negative in the HBcAg(−) group and the HBcAg(+) group

Items		HBcAg (−) (*n* = 48)	HBcAg (+) (*n* = 48)	*P*
HBV DNA‐negative rate of (%)	24 weeks	37.5 (18/48)	22.9 (11/48)	0.120
	48 weeks	77.1 (37/48)	45.8 (22/48)	0.002^a^
	96 weeks	95.3 (46/48)	83.3 (40/48)	0.045^a^
	144 weeks	100 (48/48)	93.7 (45/48)	0.242
	288 weeks	100 (48/48)	100 (48/48)	1.0
ALT normalization rate (%)	24 weeks	39.6 (19/48)	22.9 (11/48)	0.078
	48 weeks	64.6 (31/48)	43.7 (21/48)	0.041^a^
	96 weeks	85.4 (41/48)	62.5 (30/48)	0.011^a^
	144 weeks	93.7 (45/48)	72.9 (35/48)	0.006^a^
	288 weeks	95.8 (46/48)	89.6 (43/48)	0.435
HBeAg‐negative rates (%)	24^ ^weeks	2.08 (1/48)	0/48	0.315
	48 weeks	6.25 (3/48)	10.4 (5/48)	0.714
	96 weeks	35.4 (17/48)	14.6 (7/48)	0.018^a^
	144 weeks	60.4 (29/48)	20.8 (10/48)	<0.001^a^
	288 weeks	72.9 (35/48)	35.4 (17/48)	<0.001^a^
HBeAg seroconversion rate at the end of follow‐up (%)		62.5 (30/48)	29.2 (14/48)	0.001^a^
HBeAg‐negative rates in histology subgroup (%)				
Grade 1		75.0 (3/4)	12.5 (1/8)	0.067
Grade 2		72.4 (21/29)	38.7 (12/31)	0.011^a^
Grade 3		73.3 (11/15)	44.4 (4/9)	0.212
HBeAg‐negative rates in histology subgroup (%)				
Stage 0		50.0 (4/8)	17.6 (3/17)	0.156
Stage 1		87.5 (7/8)	71.4 (5/7)	0.569
Stage 2		75.0 (6/8)	22.2 (2/9)	0.057
Stage 3		71.4 (5/7)	44.4 (4/9)	0.358
Stage 4		70.6 (12/17)	50.0 (3/6)	0.621
HBeAg‐negative rates in subgroup of antiviral therapy (%)	Total			
LAM	10 (10.4)	71.4 (5/7)	100 (3/3)	1.000
ADV	11 (11.4)	71.4 (5/7)	75.0 (3/4)	1.000
LDT	4 (4.2)	0 (0/2)	100 (2/2)	0.333
ETV	54 (56.2)	78.3 (18/23)	16.1 (5/31)	<0.001^a^
LAM + ADV	14 (14.6)	87.5 (7/8)	50.0 (3/6)	0.245
ETV + ADV	3 (3.1)	0 (0/1)	50.0 (1/2)	1.000

LAM: lamivudine; ADV: adefovir dipivoxil; LDT: telbivudine; ETV, entecavir; ALT: Alanine transaminase.

HBcAb reference: 1.0000–3.000 negative and 0–1.000 positive.

HBeAg reference: <1 s/co negative and >1 s/co positive.

HBeAb reference: <1 s/co positive and >1s/co negative.

a Significant difference was found in the HBcAg(−) group and HBcAg(+) group (All *P* < 0.05).

### ALT normalization rate

The ALT normalization rate progressively increased following administration of antiviral drugs treatment in both groups. ALT normalization rates were significantly higher in the HBcAg (−) group than in the HBcAg (+) group from week 48 to 144 (63.6% *versus* 43.7% at week 48, *P* = 0.041; 85.4% *versus* 62.5%, *P* = 0.011 at week 96; 93.7% *versus* 72.9% at week 144, *P* = 0.006, Table 2). However, based on the Kaplan–Meier survival analysis, it revealed that no significant difference was found in the cumulative ALT normalization rates (*P *=* *0.058) by 288 weeks and the median survival time of ALT normalization rates were 31.0 and 54.7 weeks. (Fig. 3B).

### HBeAg‐negative rates

The frequency of HBeAg‐negative expression was significantly higher in the HBcAg (−) group than in the HBcAg (+) group from week 96 to week 288 (35.4 % *versus* 14.6% at week 96, *P* < 0.05; 60.4% *versus* 14.6%, *P* < 0.001 at week 144; 72.9% *versus* 35.4%, *P* < 0.001 at week 288; Table 2). In addition, Kaplan–Meier survival analysis revealed that the frequency of HBeAg‐negative expression showed significant difference within the two groups (*P* < 0.001) and the median survival time of HBeAg was 124 weeks in the HBcAg (−) group and 288 weeks in the HBcAg (+) group (Fig. 3).

### HBeAg‐negative rates in subgroup of histology

We grouped HBeAg (−) patients into subgroups according to histology grades (Grade 1 to Grade 3) and stages (Stage 1 to Stage 4). There was a significant difference in the Grade 2 subgroup, with 72.4% (21/29) in HBcAg (−) group and 38.7% (12/31) in HBcAg (+) group (*P *=* *0.011). No significant difference was found in the Grade 1 or Grade 3 subgroups. There was also no significant difference within any of the stage subgroups (Table 2).

### HBeAg‐negative rates in antiviral drug subgroups

Six different antiviral drug therapies were used for patients in this study (LAM, ADV, LDT, ETV, LAM + ADV or ETV + ADV). The only significant difference in HBeAg‐negative seroconversion was found in the ETV group. There was a higher frequency of HBeAg‐negative seroconversion in the HBcAg (−) group than in the HBcAg (+) group (78.3 %, 18/23 and 16.1%, 5/31, respectively), *P* < 0.001 (Table 2).

### HBcAg (−) is a predictor of HBeAg seroconversion

In the binary linear logistic regression analysis for all the factors in our study, we found that the absence of HBcAg was the only predictor for a patient being HBeAg (−) throughout the study. The Odds ratio (OR) value was 4.482 (95% CI 1.58–12.68) (Table 3).

**Table 3:** The result of binary logistic lineal regression of the HBeAg negative in all patients

Factors	B	S.E.	Wald	df	Sig.	Exp (B)	95% CI for EXP (B)	
							Lower	Upper
HBcAg (−)	1.500	.531	7.987	1	.005^a^	4.482	1.584	12.683
Sex (male)	−.076	.756	.010	1	.920	.927	.210	4.081
Age	−.294	.335	.769	1	.380	.745	.386	1.438
Antiviral drugs			7.564	5	.182			
LAM	1.741	1.585	1.206	1	.272	5.703	.255	127.459
ADV	1.604	1.576	1.036	1	.309	4.975	.227	109.237
LDT	.463	1.362	.116	1	.734	1.589	.110	22.914
ETV	.926	1.871	.245	1	.621	2.525	.065	98.744
LAM+ADV	2.507	1.563	2.573	1	.109	12.271	.573	262.613
Histology grades (G)	.298	.487	.373	1	.541	1.347	.518	3.500
Histology stages (S)	.216	.197	1.201	1	.273	1.241	.843	1.827
HBeAg baseline	−.399	.400	.996	1	.318	.671	.307	1.469
HBsAg baseline	−.304	.457	.443	1	.506	.738	.301	1.806
HBV DNA baseline	−.152	.213	.511	1	.475	.859	.566	1.303
ALT baseline	.001	.001	1.239	1	.266	1.001	.999	1.003
HBcAb baseline	2.194	2.097	1.094	1	.296	8.969	.147	546.757
Constant	−2.172	3.880	.313	1	.576	.114		

a HBcAg negative was the only significant predicator.

## Discussion

The strategy of finite‐duration treatment with NAs can be feasible for HBeAg (+) patients who seroconvert to anti‐HBe on treatment 22. However, seroconversion rates in NA‐naïve HBeAg (+) CHB patients undergoing NA treatment were low (LAM 50% 23, ADV 30–37% 24, LDT 53% 25 and ETV 26–49% 26). In our study, about 54.1% of all patients seroconverted, but 72.9% of HBcAg (−) patients became HBeAg (−) during the 288 weeks of NA therapy. These results show that HBcAg can serve as a predictor of virus response and HBeAg seroconversion.

The lack of HBcAg on IHC was a good predictor of viral response rates. We found that the viral response of the patient, as indicated by the loss of HBV DNA expression, in the HBcAg (−) group increased dramatically compared to the HBcAg (+) group from week 48 to week 96 (*P *=* *0.002, *P *=* *0.045, respectively). However, no difference was found between the two groups in week 144 (Table 2), suggesting that viral response rates were faster in the HBcAg (−) group. In addition, the frequency of HBV DNA‐negative patients was significantly higher in the HBcAg (−) group than in the HBcAg (+) group. We conclude that the rates of viral response were faster in the HBcAg (−) group compared with the HBcAg (+) group. Therefore, the absence of HBcAg in the liver as determined by IHC could be a good predictor of viral response rates.

HBcAg (−) expression correlated with more significant ALT normalization rates. Normalization of ALT levels is used as a determinant of biochemical response 22. ALT normalization rates were higher in the HBcAg (−) group than in the HBcAg (+) group from week 48 to week 144. However, there were no significant differences in cumulative ALT normalization rates in either group based on our study (Fig. 3). Therefore, these results indicate that a greater number of patients in the HBcAg (−) group recovered from liver injury and did so at a faster rate than the HBcAg (+) group. However, ALT activity often fluctuates over time and ALT normalization rates may be influenced by many other factors. Further multicenter research with more patients should be conducted to investigate the ALT normalization rates in the future.

The results of our study suggest that HBcAg (−) staining by IHC may be an important predictor of response to antiviral treatment, including HBeAg (−) rates. Our data indicated that HBeAg (−) rates were much higher in the HBcAg (−) group than the HBcAg (+) group from week 96 to week 288 (72.9% *versus* 35.4%, respectively, *P* < 0.001, Table 2). This was similar to HBeAg seroconversion rates at the end of the study (62.5% in HBcAg (−) *versus* 29.2% in HBcAg (+), *P* = 0.001). Although we separated HBeAg (−) patients from HBeAg (+) patients, we still found that the rates of HBcAg (−) patients were significantly different (Table 3). Binary linear regression results also showed that a HBcAg (−) status was the very important predictor of a HBeAg (−) status, not including ETV or histology grade (Table 3). There are several reasons for how HBcAg (−) on IHC could predict the HBeAg (−) expression. First, HBcAg is one of the hepatitis B viral proteins 27 considered an indicator of active viral replication, especially as HBcAg expression patterns in hepatocytes had been found to be related to the activity of liver disease, hepatocyte proliferation and HBV DNA level 28. As a result, a HBcAg (−) status means that there is less active viral replication and therefore lower propensity to make HBeAg. Second, HBcAg (−) status indicates a more active T cell response against HBV and thus results in less HBeAg expression. HBcAg expression in the nucleus is lower in patients with more active hepatitis B than in patients with inactive CHB 29, 30. HBcAg can shift from the nucleus to the cytoplasm when cells undergo division after liver damage and HBcAg may be lower or lost in the nucleus with further reproduction of HBV DNA. Third, the presence of HBcAg in the hepatocyte nucleus may be positively correlated with intrahepatic cccDNA level. Lack of HBcAg in the hepatocyte indicates that the cccDNA could be very low or the supply for cccDNA pool could be deficient. Therefore, patients with a HBcAg (−) status during NA therapy may have better outcomes. This result was consistent with research by Uzun *et al*. 31, who also found that absence or a low level of HBcAg expression in the liver seemed to predict a patient's response to antiviral treatment. Our results were also consistent with research by Lee *et al*. 14 that showed that patients with CHB with a HBcAg (−) status their hepatocyte nuclei had a better response to ETV. Taken together, we found that the absence of HBcAg in the hepatocyte nuclei on IHC could be a good predicator for HBeAg (−) expression in HBeAg (+) patients with CHB.

However, there are some limitations of this retrospective study. First, all the patients were enrolled at one institute, reducing the diversity of the population studied. Second, patients did not receive the same treatment of antiviral drugs, although most had ETV and even within this subgroup, we found an apparent difference between the two groups. We will continue to collect data from the ETV‐ and TDF‐treated patients who are HBcAg (−). Finally, the genotype of HBV was not measured in the study. It has been reported that treatment responses rates to different NAs (including LAM, ADV, LDT and ETV) are similar in most HBV genotypes 32, 33. In the future, to validate the value of the HBcAg expression status in hepatocytes for predicting response to NAs, further research on the mechanisms with which HBcAg is involved in the hepatocyte nucleus is needed. However, based on our rigorous analysis and investigation, we strongly believe that HBcAg (−) status on liver biopsy can be considered a good predictor for HBeAg seroconversion rates in HBeAg (+) patients with CHB.

In conclusion, lack of HBcAg in the hepatocyte nucleus on IHC of a liver biopsy could be an independent predictor for likelihood of HBeAg seroconversion during in NA‐naïve patients with CHB who are HBeAg (+). The function and underlying mechanisms of the HBcAg in the hepatocyte nucleus during NA therapy in patients with CHB need to be further investigated.

## Conflict of interest

The authors declare that they have no competing interests.

## Author contributions

J.X., X.L., M.H. conceptualized the study. M.H. and J.L. conducted the data analysis, interpreted results and wrote the manuscript. M.C., X.Z., Z.H., Z.H., J.X. and Z.Z. performed the fieldwork and data collection. M.H. and J.L. performed the data management. X.L. and J.X. contributed to interpretation of the results and the manuscript writing. All authors read and approved the final manuscript.

## Ethics approval and consent to participate

This study was approved by the 5th Affiliated Hospital Ethical Committee at SYSU. Written consent to participate in the study was obtained from each participant in the study.
